# Meniscal ramp lesions: diagnostic performance of MRI with arthroscopy as reference standard

**DOI:** 10.1007/s11547-021-01375-3

**Published:** 2021-06-03

**Authors:** Marcello Zappia, Luca Maria Sconfienza, Salvatore Guarino, Michele Tumminello, Germano Iannella, Pier Paolo Mariani

**Affiliations:** 1 Department of Medicine and Health Sciences, University of Molise, Campobasso, Italy; 2 Varelli Institute, Naples, Italy; 3 IRCCS Istituto Ortopedico Galeazzi, Milan, Italy; 4 Department of Biomedical Sciences for Health, Università Degli Studi di Milano, Milan, Italy; 5 Department of Radiology, Monaldi Hospital, Naples, Italy; 6 Department of Economics, Business and Statistics, University of Palermo, Palermo, Italy; 7 Villa Stuart Sport Clinic, FIFA Medical Centre of Excellence, Rome, Italy; 8 Department of Movement, Human and Health Sciences, University of Rome Foro Italico, Rome, Italy

**Keywords:** MRI, Arthroscopy, ACL injuries, Meniscus, Anatomy

## Abstract

**Background:**

The posteromedial meniscal region is gaining interest among orthopedic surgeons, as lesions of this area has been reported to be significantly associated with anterior cruciate ligament tears. The current imaging literature is unclear.

**Purpose:**

To evaluate the diagnostic performance of MR in the detection of meniscal ramp lesions having arthroscopy as reference standard.

**Materials and methods:**

We retrospectively included 56 patients (mean age of 25 ± 7 years; 14 females) from January to November 2017 with a arthroscopically proved ACL tear and posterior meniscocapsular separation. On preoperative MRI, two radiologists with 13 and 2 years’ experience in musculoskeletal imaging assessed the presence/absence of ramp lesion, meniscotibial ligament lesion, peripheral meniscal lesion, or their combination, bone bruise. Having arthroscopy as reference standard, diagnostic performance of MRI in the evaluation of ramp area lesions was calculated. Cohen’s kappa (*k*) and Fisher's Exact Test statistics were used.

**Results:**

Agreement between radiologists ranged from *κ* = 0.784 (meniscotibial ligament lesions) to κ = 0.918 red–red meniscal lesion. Sensitivities were 97.4% for ramp lesions, 95.8% for meniscotibial ligament lesion, 94.4% for peripheral meniscal lesions; specificities were 88.9%, 81.3%, and 97.4%, respectively; accuracies were 94.6%, 87.5%, and 96.4%, respectively. Agreement between MR and arthroscopy was almost perfect in identification of ramp lesions (*κ* = 0.871) and red–red zone meniscal lesions (*κ* = 0.908). The agreement between the two methods was substantial (*κ* = 0.751) for meniscotibial lesion. No significant association between tibial plateau bone bruise and the different type of lesions was found (*κ* ≥ 0.004 and *p* ≥ 0.08).

**Conclusion:**

MR has high diagnostic performance in meniscal ramp area lesion assessment, with substantial to almost perfect inter-reader agreement.

## Introduction

The posteromedial region of the meniscus is gaining increasing interest among orthopedic surgeons, as lesions of this area have been reported to be significantly associated with anterior cruciate ligament tears, with implications for patients’ care [1]. In particular, Strobel et al. described a specific type of tear, involving the peripheral attachment of the posterior horn of medial meniscus, currently referred to as *ramp lesions* [2].

**Fig. 1:**
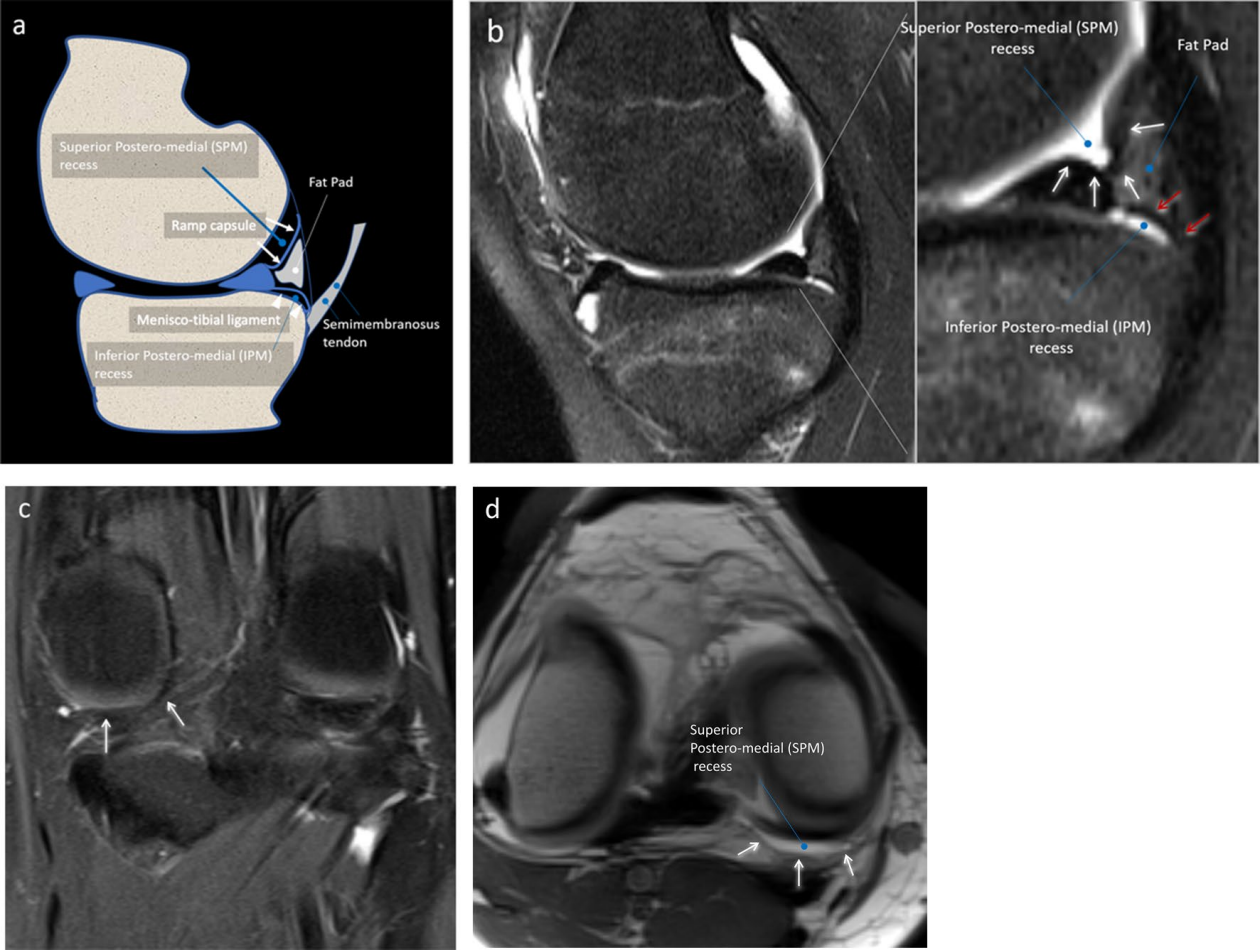
**a–d** Drawing **a **PDw fat sat sagittal **b** and coronal **c** MR images and T1w MR-arthrography image show the ramp capsule (white arrows) forming the floor of the superior posteromedial recess (black asterisks). The meniscotibial (or coronary) ligament (white arrowheads) forms the roof of a second and different recess that we have called the inferior posteromedial recess (black hashtags). A small and extra-articular fat pad is present posteriorly to both recesses (white asterisk)

Strobel defined the *ramp* area as capsular reflection, arthroscopically visible by the superior posteromedial (SPM) recess of the knee [3]. That recess is delimited superiorly by the medial femoral condyle, inferiorly by the superior part of the posterior meniscal horn, and posteriorly by the ramp capsule [3, 4]. During arthroscopy, which is performed with the knee 90° flexed, the ramp thin capsule is deflected, and the SPM recess widens. However, magnetic resonance imaging (MRI) is routinely performed with the knee completely extended (or few grades flexed) and, therefore, the SPM recess is very small. On MR sagittal images, in this area another different recess can be seen, which has never been reported before, and that can be defined as inferior posteromedial (IPM) recess. That recess is delimited postero-superiorly by the posterior part of meniscotibial (or coronary) ligament and anteriorly from the tibial plate [5–7] Fig. 1a, b. In normal conditions, the IPM recess cannot be accessed with arthroscopy and the SPM and IPM recesses do not communicate [3]. If MRI is performed with knee 90° flexed, the SPM recess widens and the IPM recess collapses, similarly to what happens during arthroscopy. Last, the SPM and IPM recesses, then the ramp capsule and the meniscotibial ligament, are normally separated by a thin fat pad [4] and Fig. 1a–d, a detail which has been neglected in the orthopedic literature, probably because during arthroscopy this area is covered and dislocated by the capsule. At any rate, a detailed knowledge of this anatomic area is crucial, as the ramp region was previously often mismatched with the IPM recess and the meniscotibial region [1].

Peripheral lesions of the posteromedial meniscus-capsular region may occur in up to 40% of anterior cruciate ligament (ACL) injuries [8]. Patients with an ACL tear associated with a medial meniscus ramp tear showed a greater amount of dynamic rotational laxity compared to patients with isolated ACL tear and no ramp tear [9]. Furthermore, the meniscotibial ligament helps the meniscus stability, providing a brake stop function to the anterior translation of the tibia. The absence of this ligament could increase joint stress and risk of injury to the chondral tibial surface and meniscus itself [10].

During arthroscopy for ACL reconstruction, ramp lesions located at posteromedial blind spot may be missed using classic standard anterolateral and anteromedial arthroscopic portals [2, 11]. While additional arthroscopic views and portals, such as intercondylar view and posteromedial portal, are extremely accurate in detecting and repairing these lesions [8, 12–14], they are not routinely used.

It is our opinion that a precise preoperative identification of each damaged structure of the ramp area may allow for a more precise preoperative planning and understand the pathogenetic mechanisms underlying meniscal instabilities.

Several previous papers on the MR evaluation of posteromedial meniscocapsular lesions have been published [15–17]. However, most of them belong to the orthopedic literature and are unclear in terms of anatomy, MR technique, and image readers. Also, a clear MR classification of ramp area lesions has never been established. Thus, our purpose was to evaluate the diagnostic performance of MR in the detection of isolated and/or combined lesions of meniscal ramp capsule, meniscotibial ligament and peripheral meniscus, having arthroscopy as reference standard. Moreover, the second aim is to evaluate the association of each lesion with meniscal instability.

## Materials and methods

Local Institutional Review Board approved this retrospective study. Patients included in the present study provided written consent for anonymized data usage for research purpose at the moment of MR examination. Institutional Review Board accepts this consent as informed consent for the present study. After matching imaging and surgical data, our database was completely anonymized to delete any connections between data and patients’ identity according to the current General Data Protection Regulation. This paper has been drafted following the Standards for Reporting Diagnostic accuracy studies (STARD) checklist.

### Study population

**Fig. 2:**
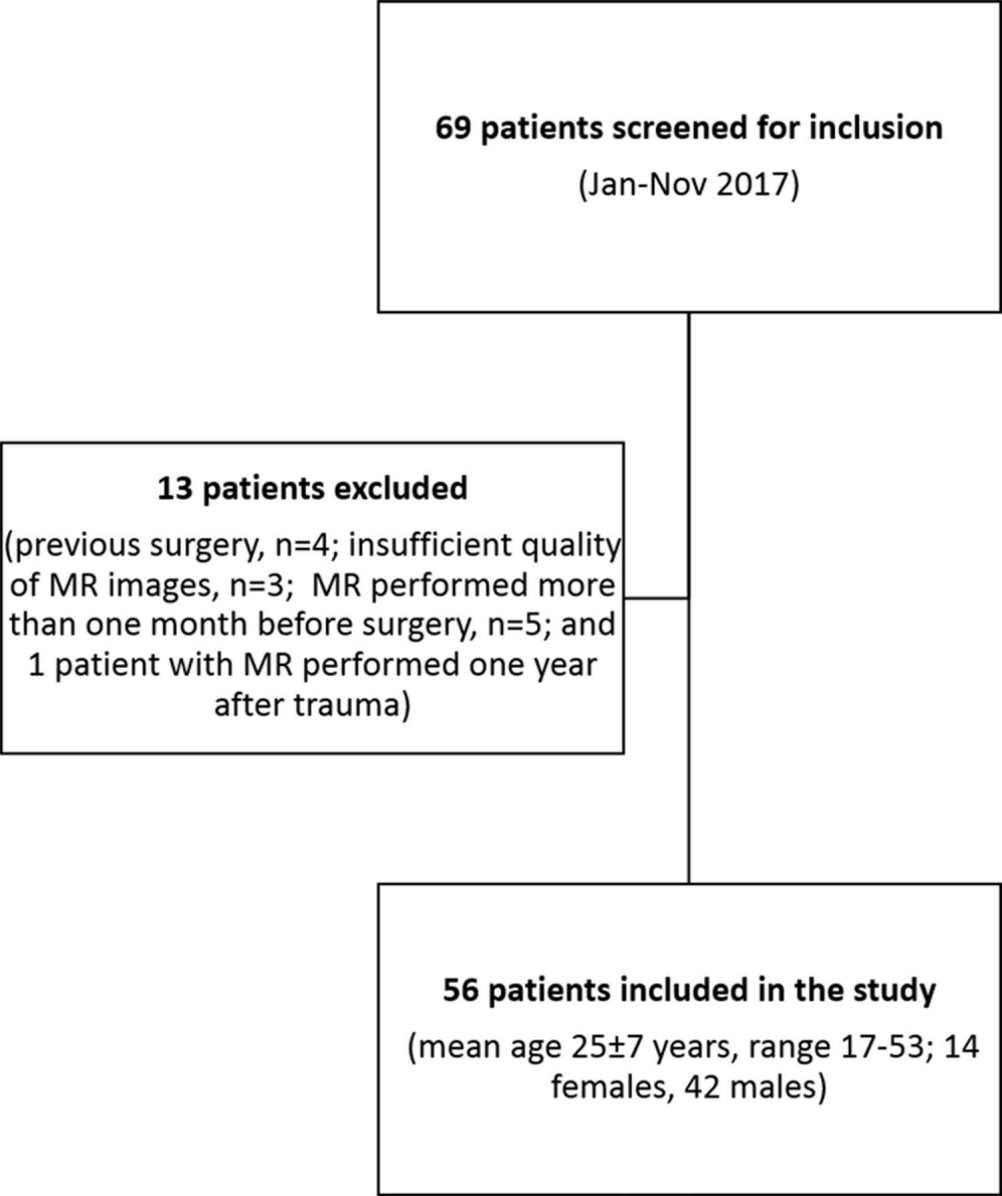
Sagittal T1w MR-Arthrography image. With knee flexed at 90° the deflexed ramp capsule (arrows) and a wide SPM recess (asterisks) can be noted as well as arthroscopy and IPM appears collapsed. SPM – Superomedial recess; IPM – Inferomedial recess

Sixty-nine consecutive patients admitted at our Institution from January to November 2017 with an arthroscopically proved ACL tear and posterior meniscocapsular separation were retrospectively screened to be included in our series. Further inclusion criteria were the availability of an MR examination performed not longer than 6 months after trauma and within one month prior to surgery. ACL tears were diagnosed at MRI and confirmed during arthroscopic treatment. Out of 69 patients, 13 patients were excluded (previous surgery, *n* = 4; insufficient quality of MR images, *n* = 3; MR performed more than one month before surgery, *n* = 5; and 1 patient with MR performed one year after trauma). Thus, our final study population included 56 patients with mean age of 25 ± 7 years (age range 17–53 years; 14 females, mean age of 23 ± 8 years and 42 males, mean age of 25 ± 6 years). A flowchart of the study is reported in Fig. 2.

All MR examinations were performed at 1.5 T with dedicated phase-array coils, with fully extended knee, at our Institution or outside. All examinations had available at least one proton density (PD) or intermediate-weighted (IW) sequence with fat saturation and one T1-weighted or PD on sagittal plane; slice thickness was ≤ 4 mm; matrix ≥ 256 × 256; field of view ≤ 160 mm.

### Arthroscopic procedure and analysis

**Fig. 3:**
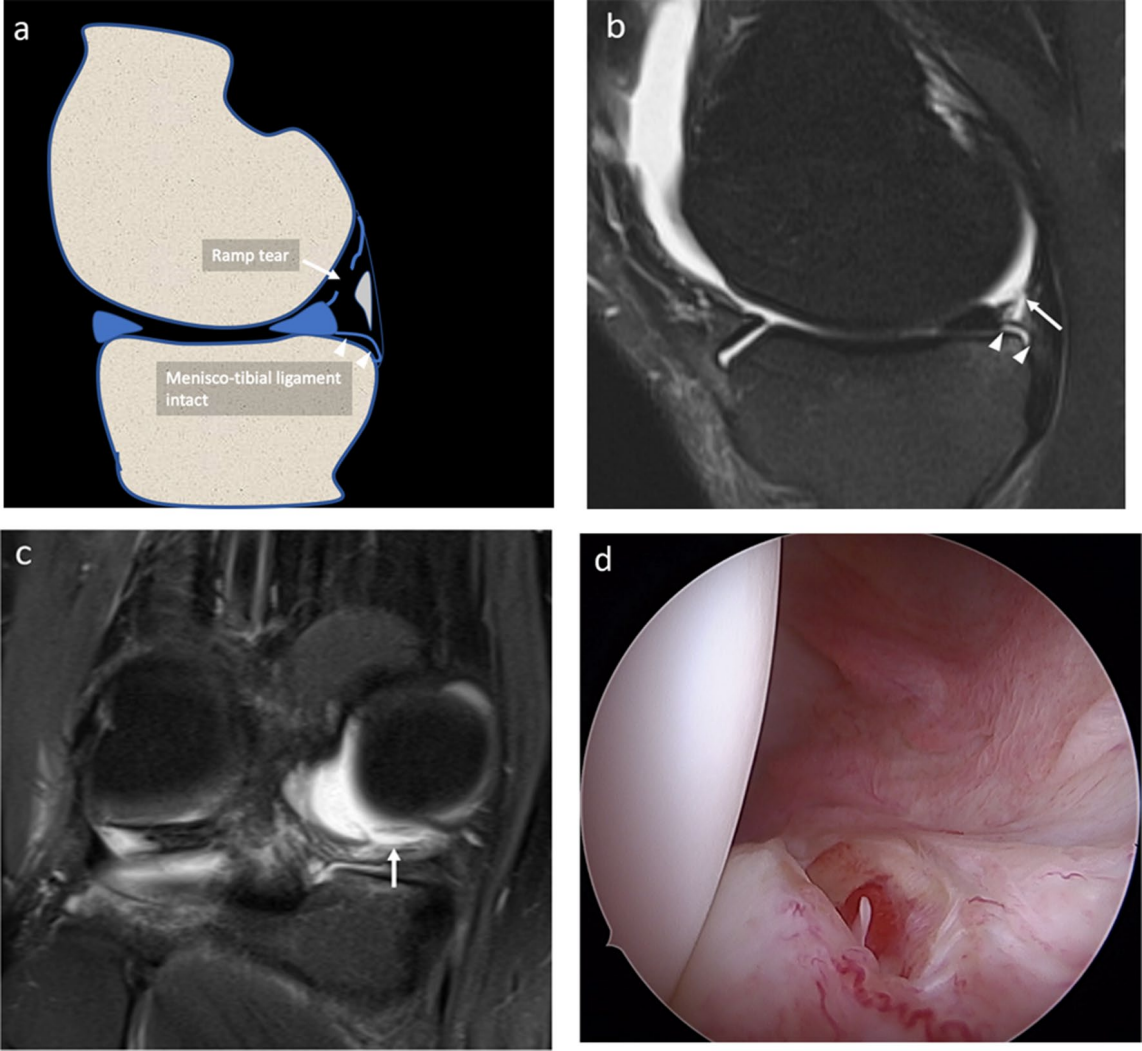
**a–d** Drawing **a**, PDw fat sat sagittal **b** and coronal **c** MR and arthroscopy **d** images of the same patient, show the ramp area lesion (arrows). The meniscotibial ligament appears intact (arrowheads)

**Fig. 4:**
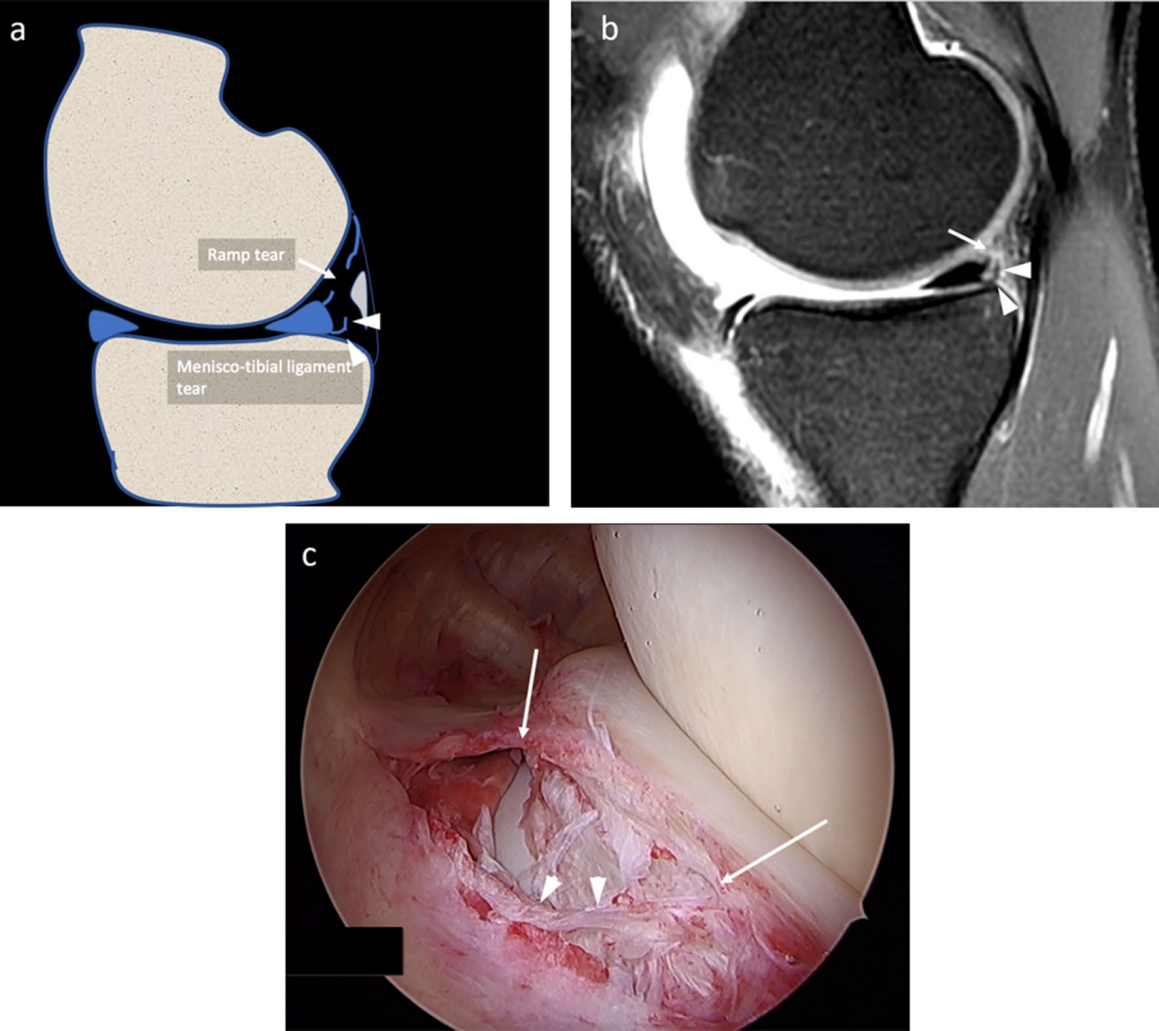
**a–c** Drawing **a**, PDw fat sat sagittal MR **b** and arthroscopy **c** images of the same patient, show a wide ramp lesion (arrows). The meniscotibial ligament appears avulsed (arrowheads) and a tibial plate was uncovered and visible by the arthroscopic approach

**Fig. 5:**
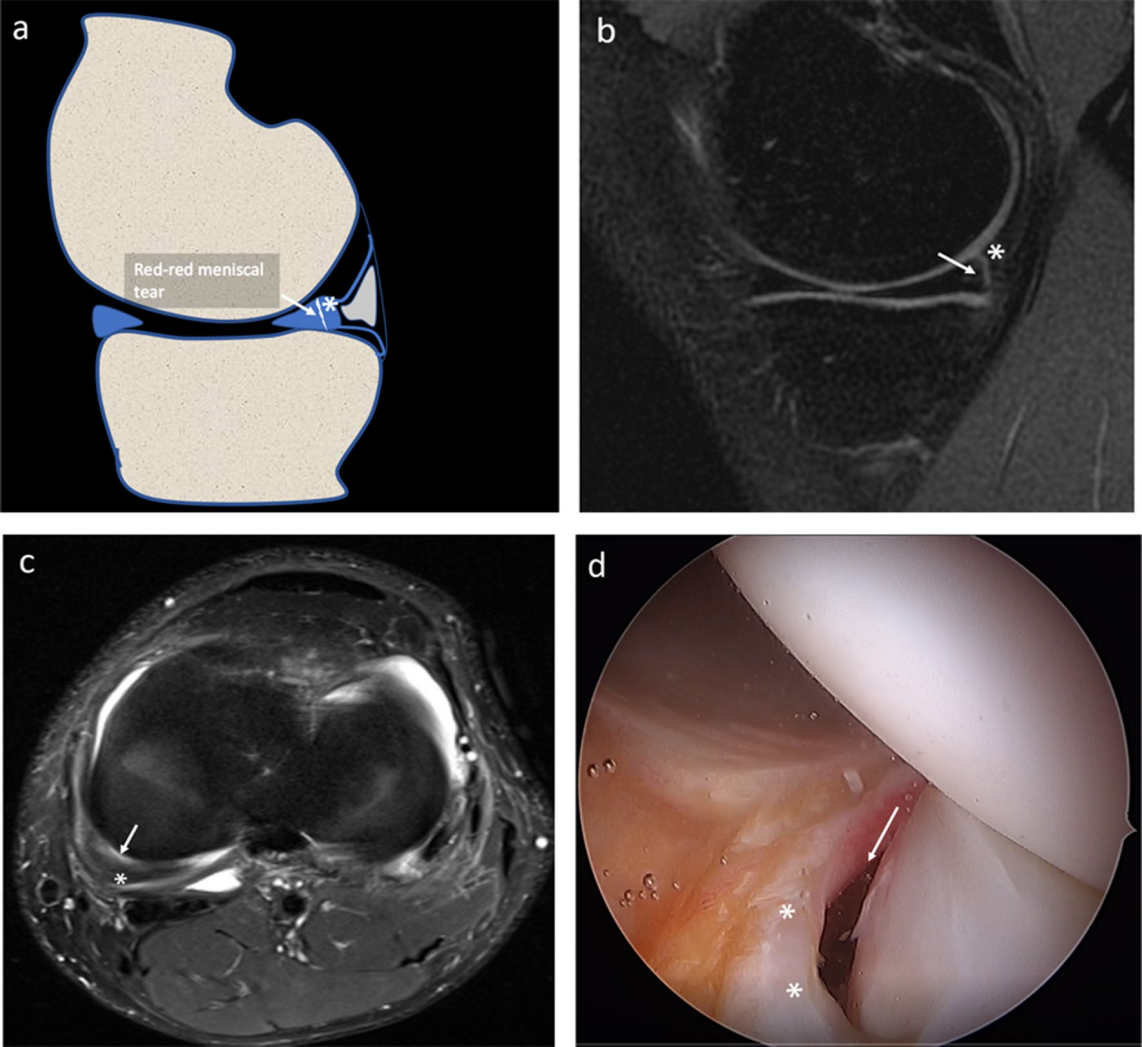
**a–d** Drawing **a**, PDw fat sat sagittal **b** and axial MR **c** and arthroscopy **d** images of the same patient, show a peripheral meniscal vertical lesion (arrow), with a small meniscal fragment (asterisks) attached to the ramp

All arthroscopic procedures were performed by a senior orthopedic surgeon (MPP), with 40 years’ experience in knee surgery. During arthroscopy, patients were positioned supine on the operating table. A foot support was used to allow positioning with the knee in 90° flexion and to allow free movements of the leg. A lateral support was positioned at the level of the tourniquet. The standard lateral parapatellar portal was used and the whole procedure was carried out with a 45° optics. After inspection of anterior compartments, the posteromedial compartment was inspected using an intercondylar notch view with the patient’s knee held in 90° flexion. The meniscocapsular junction was routinely probed with a spinal needle. If a hidden lesion was suspected, minimal debridement of the synovial sheath was performed. The knee is flexed and extended several times and an additional posteromedial portal was performed in the presence of ramp lesion to inspect the lesion. The ramp area was probed and grasped to examine the meniscotibial ligament. Furthermore, the meniscus was probed during movement of the knee to evaluate its stability. All procedures were recorded on digital supports and stored. Three months after the last procedure and blinded to MR findings, the same orthopedic surgeon revised the videos of the procedures and posteromedial meniscocapsular lesions were defined as follows:

- A ramp lesion was identified when the medial ramp capsule was interrupted or when the posterior margin of the capsule presented a laxity covered by inhomogeneous synovial tissue [3] Fig. 3a–d.
- A lesion of the meniscotibial ligament was identified when the ligament was interrupted and a portion of the posteromedial tibial plate was visible from the SPM recess through both the ramp area tear and meniscotibial ligament tear Fig. 4a–c.
- A vertical–longitudinal peripheral meniscal lesion was identified as an interruption of the peripheral part of the posterior horn of meniscus with a meniscal fragment still attached to the ramp capsule Fig. 5a–c.

A lesion was defined unstable if during knee flexion and extension the meniscus was mobile making a portion of the posteromedial tibial plate visible.

### MR image analysis

Two radiologists (MZ and SG, with 13 and 2 years’ experience in musculoskeletal imaging, respectively), blinded to orthopedic assessment, independently assessed MR images. They were asked to assess each structure of the posteromedial meniscocapsular complex on sagittal PD or IW fat saturated sequences. Criteria for diagnosing tears of those structures were:

- Ramp lesion: when the concave band (ramp capsule) with low signal intensity posterior to the posterior horn of the medial meniscus was interrupted by a line of hyperintensity crossing this capsular reflection Fig. 3a–c;
- Meniscotibial ligament lesion: when the ligament appeared interrupted and hyperintense, with a fluid line extending close to the posterior margin of tibial plate, having an acute angle appearance while having lost the normal curved and concave shape Fig. 4a–c;
- Peripheral meniscal lesion: when a vertical or oblique hyperintense line was identified in the posterior third of the posterior horn of medial meniscal fibrocartilage (1–3 mm from the meniscocapsular junction) Figs. 5a–c

**Fig. 6:**
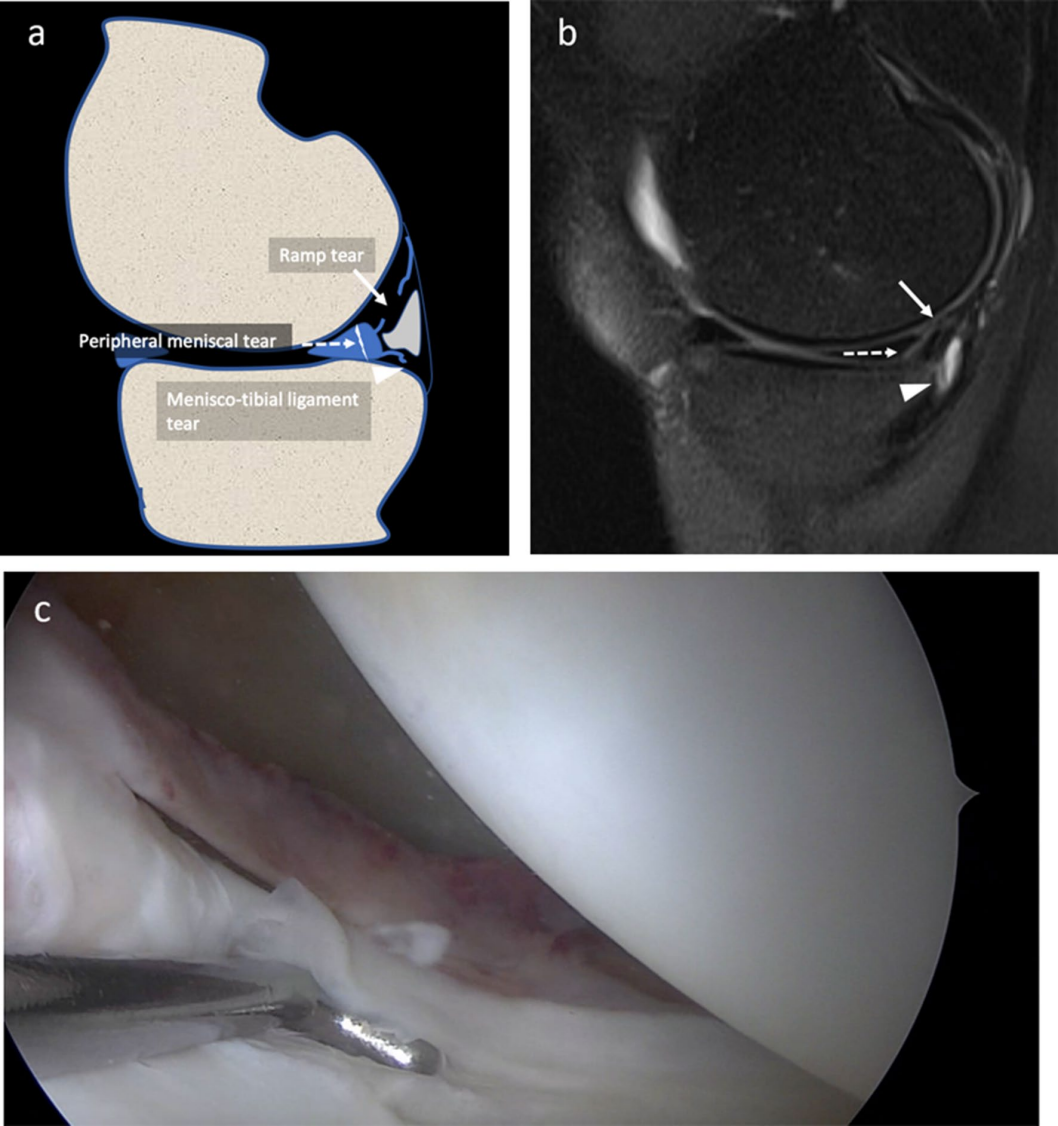
**a–c** Drawing **a**, PDw fat sat sagittal MR **b** and arthroscopy **c** images of the same patient, show the association of more lesions. Ramp lesion (arrow), peripheral meniscal oblique lesion (dotted arrows), and meniscotibial ligament tear (arrowheads)

The association of more type of lesions was assessed Fig. 6a–c. The presence of bone bruise edema of the posteromedial tibial plate was also assessed. Lesions were graded as present or absent.

### Statistical analysis

Cohen’s kappa (*k*) was used to evaluate the agreement between the two radiologists in the identification of the lesion of the single structure and to evaluate the agreement between sagittal PD or IW MR sequences assessed by the most experienced radiologist and arthroscopy in the identification of the lesion of each structure.

Having arthroscopy as reference standard, sensitivity, specificity, positive predictive value, negative predictive value, and accuracy of MR in diagnosing ramp lesions, meniscotibial ligament lesions, and peripheral fibrocartilage meniscal tears were given as percentage with their 95% confidence intervals calculated according the exact binomial distribution.

The agreements between MR findings reported by the most experienced radiologist and arthroscopy in identification for each structure damaged and in classification were calculated.

Cohen’s kappa (*k*) and Fisher’s exact test were used to evaluate the association between the type of damaged structure at MR assessed by the most experienced radiologist and arthroscopic meniscal instability or tibial plate bone bruise at MR.

Statistical significance was set at *p* < 0.05. The SPSS software (v. 26, IBM, Armonk, New York, NY) was used for statistical analysis.

## Results

The agreement between the two radiologists was almost perfect for the detection of ramp lesions (*κ* = 0.908) and red–red meniscal lesion (*κ*  = 0.918). For meniscotibial ligament lesions, the agreement was substantial (*κ* = 0.784).

Full data of MR diagnostic performance having arthroscopy as reference standard are reported in Table 1. Accuracy was 94.6% for ramp lesions, 87.5% for meniscotibial ligament lesions, and 96.4% for peripheral zone fibrocartilage meniscal lesions.

**Table 1:** Diagnostic performance of magnetic resonance imaging in the diagnosis of ramp lesions, meniscotibial ligament lesions, peripheral fibrocartilage meniscal lesions, having arthroscopy as reference standard in a series of 56 patients

	Ramp lesion	Meniscotibial ligament lesion	Peripheral meniscal lesion
True positive	*n* = 37	*n* = 23	*n* = 17
False positive	*n* = 2	*n* = 6	*n* = 1
False negative	*n* = 1	*n* = 1	*n* = 1
True negative	*n* = 16	*n* = 26	*n* = 37
Sensitivity	97.4% (86.2–99.9%)	95.8% (78.9–99.9%)	94.4% (72.7–99.9%)
Specificity	88.9% (65.3–98.6%)	81.3% (63.6–92.8%)	97.4% (86.2–99.9%)
PPV	94.9% (82.7–99.4%)	79.3% (60.3–90.2%)	94.4% (72.7–99.9%)
NPV	94.1% (71.3%–99.9%)	96.3% (81.0%–99.9%)	97.4% (86.2%–99.9%)
Accuracy	94.6% (85.1–98.9%)	87.5% (75.9–94.8%)	96.4% (87.7–99.6%)

Note: 95% confidence intervals are indicated in brackets. *PPV* positive predictive value; *NPV* negative predictive value

The agreement between MR and arthroscopy was almost perfect in identification of ramp lesions (*κ* = 0.871) and red–red zone meniscal lesions (*κ* = 0.908). The agreement between the two methods was substantial (*κ* = 0.751) in the identification of meniscotibial lesion. The MR and arthroscopy classifications is summarized in Table 2. The agreement of two methods for classification reached 84% (47/56).

**Table 2:** MR and arthroscopy classifications

	MR	Arthroscopy
Isolated ramp lesion	*n* = 14	*n* = 17
Isolated MTL lesion	*n* = 0	*n* = 0
Isolated peripheral meniscal lesion	*n* = 13	*n* = 14
Ramp + MTL Lesion	*n* = 24	*n* = 20
Peripheral meniscal + MTL lesion	*n* = 3	*n* = 2
Ramp + peripheral + MTL lesion	*n* = 2	*n* = 2

Note: *MTL* meniscotibial ligament

Regarding meniscal instability, meniscotibial ligament lesions at MR were present in 21/22 (95.5%) patients with arthroscopic instability and in 8/34 (23.5%) patients without arthroscopic instability with substantial agreement (*κ* = 0.681) and *p* < 0.001. In one patient, no clear lesions were found at arthroscopy except for meniscal instability. At MR, ramp and meniscotibial ligament lesions were found.

Bone bruise was seen in 29/56 (51.8%) patients at MR. Of them, 17/29 (55.2%) patients had also a ramp lesion, 15/29 (51.7%) patients had meniscotibial ligament lesions, and 13/29 (44.8%) patients had peripheral fibrocartilage meniscal lesions. No significant association between tibial plateau bone bruise and the different type of lesions was found (ramp lesion *κ* = 0.223 and *p*-value = 0.0843; meniscotibial ligament lesion *κ* = 0.004 and *p*-value = 1; peripheral meniscal lesion *κ* = 0.223 and *p*-value = 0.0949).

## Discussion

Our main findings are that MRI has high diagnostic performance in the evaluation of meniscal ramp area lesions with substantial to almost perfect inter-reader agreement. Also, lesions of the meniscotibial ligament are significantly associated with meniscal instability as dynamically assessed at arthroscopy, while posteromedial tibial plate bone bruise is not associated with any specific ramp area lesions.

Sonnery-Cottet et al. [8] and Thaunat et al. [13] classified the medial meniscocapsular tears into the following five types: ramp lesion, partial superior lesion, partial inferior or hidden lesion, complete tear, and double tear. [8]. Greif et al. in their MRI review study proposed the Thaunat classification [18]. In a recent study the arthroscopy classification for meniscal ramp lesions stability adapted for MRI has shown good reproducibility when applied by trained musculoskeletal radiologists [19]. The most relevant difference between arthroscopy and imaging is that the arthroscopy is performed with the knee flexed while MR is performed with the knee extended. The discrepancy between anatomical drawing of Sonnery-Cottet et al. [8] and MRI anatomical findings that we have described is due to not including the thin fat pad between the ramp capsule and the meniscotibial ligament, clearly recognizable on sagittal MR images, and to the fact that to few grades of knee flexion during MR exam the ramp capsule is collapsed and the SPM recess is smaller than in arthroscopy. For this reason, we preferred to evaluate the damage of each anatomical structures, also to understand their biomechanical role of them.

Regarding meniscal instability, our data agree with Sonnery-Cottet et al. and Thaunat et al., who report association between meniscal instability and meniscotibial ligament lesions. Differently from Thaunat et al., we may hypothesize that MR could be superior to arthroscopy in the identification of meniscotibial ligament tears. If ramp capsule is intact or covered by fibrous reparative tissue, meniscotibial ligament lesions (also known as “hidden lesion” [13]) cannot be visualized from SPM recess during arthroscopy and, to our knowledge, the arthroscopy of the IPM recess have never been described. However, this represents a mere speculation which cannot be directly derived from available data.

MR accuracy in detecting ramp lesions was discussed in previous studies with very conflicting results. However, most studies are unclear in terms of methodology: they do not state which magnetic field strength [15, 16] or which sequences were used [11, 15], who read the MR images, with which experience [11, 15, 17] and with which criteria [15, 16], or which statistical analysis was carried out [16]. With these non-negligible limitations, Malatray et al. [16] reported that sensitivity of MR to diagnose ramp lesions is very low, while Arner et al. found moderate to moderate-high sensitivity and excellent specificity in detecting meniscal ramp lesions at MR, suggesting excellent accuracy in identifying ramp lesions but slightly lower accuracy in detecting all ramp injuries. [11].

Yeo et al., in a retrospective study including seven ramp lesions detected on arthroscopy in patient with ACL tear, reported that irregularity of the posterior margin of the posterior horn of medial meniscus and complete fluid filling between the meniscus and the capsule margin were the most sensitive findings for detecting ramp lesion on MRI [20]. The low number of patients enrolled was a significant weakness point of their study. Rubin et al. report that positive predictive value for meniscocapsular separation at MR is low, but the contrast and spatial resolution of MR images today have been definitely improved compared to 1996 [5]. The most complete work on the topic is by Hatayama et al., who reported 71.7% sensitivity and 90.5% specificity for the MR diagnosis of ramp lesion, with no significant differences between 1.5 and 3 *T* [14]. While specificity is in line with our data, we found a sensitivity value about one-third higher than them. One explanation of this difference may be a different interval between trauma and MR examination (not reported by Hatayama et al.): in our series, all patients underwent MR examination not longer than 6 months from trauma and the presence of fluid may have helped the diagnosis. However, even though we did not calculate the isolated diagnostic performance, when fluid was absent the obliteration of retromeniscal fat pad was used as helpful sign of ramp lesion.

We agree with the theory that posteromedial tibial plate bone bruise may be due to traction or avulsion of meniscotibial ligament and/or semimembranosus tendon [21] but we did not found the expected association between bone bruise and meniscotibial ligament lesion. DePhillipo et al. reported that bone bruise was identified on preoperative MR exam in 72% of all patients with a combined ACL tear and medial meniscal ramp lesion, suggesting to be an important secondary finding of ramp lesion [14]. Kumar et al. also found that bone bruise correlated with peripheral tears [15], in patients with bone bruise on preoperative MRI, who exhibited 2.1 times greater odds of a diagnosis of a ramp lesion compared with more central meniscal body tears. However, our findings were more in accordance with Hatayama et al. who reported the incidence of bone bruise was not significantly different among ramp lesions (38.5%), meniscal body tears (40.0%), or no tears (30.5%) in ACL-injured knees [10], being their percentage of bone bruise seen in ramp lesion similar to ours. Kaplan et al. addressed the posteromedial tibial plateau bone edema to a contrecoup injury mechanism. They hypothesized that an impaction between the medial femoral condyle and the tibial plateau during varus stress trauma determined the bone bruise [22]. Further studies are warranted to explain the exact mechanism of posteromedial tibial plateau bone bruise in patients with ramp lesions.

The terms *meniscocapsular separation* and *ramp lesion* were used indistinctly in the literature. Actually, we think the ramp lesion is only a part of meniscocapsular separation for two reasons. First, the meniscocapsular separation could involve also the meniscal body, where meniscocapsular anatomy is different and where there is no clear cleavage plane between the capsule and the meniscus. The structure covering the meniscal body is named the menisco-femoral ligament, which represents the extension of deep portion of medial collateral [4]. Thus, a meniscocapsular separation localized to the body of meniscus determines a real separation of meniscus and capsule, since these lesions could be hidden to arthroscopy and it is important to differentiate them from those of the posteromedial region. Second, in posteromedial region, the ramp capsule is only one of several structures that provide the stability of the meniscus, and thus, a posteromedial meniscocapsular separation can involve or not the ramp capsule. Moreover, the ramp capsule alone seems not to be crucial in meniscal stability, while meniscotibial ligament and semimembranosus tendon may have a stronger implication [23].

Limitations should be considered. First, this is a retrospective study. However, all data were available for review during the retrospective analysis. A prospective evaluation may clarify a superiority of MR in the evaluation of those hidden lesions missed at arthroscopy for the presence of scarring tissue. Then, MR examinations were performed at different hospitals with different scanners. However, all examinations were performed at 1.5 *T* and adequate sequences were always available for review. The major limitation of the study is that our population only included patients with posteromedial meniscocapsular tears and we do not have a healthy control group, although as control cases we used the subjects with a different type of posteromedial lesion than the one analyzed and this allowed us to calculate specificity, negative predictive value, and accuracy. Thus, the reliability of these findings in a clinical routine basis is yet to be demonstrated.

In conclusion, MR has high diagnostic performance in meniscal ramp area lesion assessment, with substantial to almost perfect inter-reader agreement. Lesions of the meniscotibial ligament are significantly associated with meniscal instability.

## Abbreviations

SPM: Superior posteromedial
MRI: Magnetic resonance imaging
IPM: Inferior posteromedial
ACL: Anterior cruciate ligament
STARD: Standards for reporting diagnostic accuracy studies
PD: Proton density
IW: Intermediate-weighted
